# Skeletal muscle extramedullary plasmacytoma transformed into plasmablastic plasmacytoma: a case report

**DOI:** 10.1007/s00432-023-05604-2

**Published:** 2024-02-01

**Authors:** Shuang Zhang, Zheng Zhi, Jie Yang, Shumin Cao, Nan Wu, Lin Kang, Jing Zhao

**Affiliations:** 1 Department of Oncology, Hebei General Hospital, NO.348 Heping West Road, Xinhua District, Shijiazhuang City, 050051 Hebei Province People’s Republic of China; 2 Graduate School, Hebei North University, Zhangjiakou, 075000 China; 3 Department of Basic Medicine, Hebei University of Chinese Medicine, Shijiazhuang, 050200 China; 4 Department of Hebei Province Chinese Medicine, Shijiazhuang, 050013 China; 5 Department of Hematology, Hebei General Hospital, Shijiazhuang, 050051 China; 6 Graduate School, Hebei Medical University, Shijiazhuang, 050017 China; 7 Department of Pathology, Hebei General Hospital, Shijiazhuang, 050051 China

**Keywords:** EMP, Plasmablastic, Gastrocnemius, Radiotherapy, Plasmacytoma

## Abstract

**Background:**

Extramedullary plasmacytoma (EMP) is a rare plasma cell malignancy, especially when the tumor originates in skeletal muscle. Plasmablastic plasmacytoma is an anaplastic round cell tumor with highly malignancy and poor prognosis. To date, there have been no reports on the transformation of skeletal muscle EMP into plasmablastic plasmacytoma. Therefore, the diagnosis, treatment, and prognosis of cases of this pathologic transformation are unclear.

**Case presentation:**

This article reports a case of an elderly male patient who presented with a painless mass in the right calf and was diagnosed with EMP by puncture pathology. Complete remission was obtained after sequential chemoradiotherapy. 6 months later, another puncture was performed due to plasmablastic plasmacytoma multiple distant metastases, and the pathology showed that EMP was transformed to plasmablastic plasmacytoma. Despite aggressive antitumor therapy, the disease continued to deteriorate, and the patient ultimately died of respiratory failure.

**Conclusion:**

The transformation of EMP into plasmablastic plasmacytoma is very rare, and its diagnosis and treatment require the participation of both experienced pathologists and clinicians. We report this case in order to raise clinicians' awareness of the diagnosis and treatment of EMP and its transformation to plasmablastic plasmacytoma, and to avoid misdiagnosis and underdiagnosis.

## Introduction

Extramedullary plasmacytoma (EMP) is a rare clonal plasma cell disease that accounts for 3–5% of all plasma cell tumors (Holler et al. 2022). 80–90% of EMPs occur in the head and neck region, followed by the gastrointestinal tract, and rarely in skeletal muscle. The prognosis of EMP is relatively good compared to solitary bone plasmacytoma (SBP) and multiple myeloma (MM), but 20–30% will eventually transform into MM (Zhu et al. 2020). MM is a malignant plasma cell clonal disease characterized by the secretion of large amounts of monoclonal immunoglobulin, which is mainly manifested by hypercalcemia, renal impairment, anemia, and bone destruction. To date, there have been no reports of transformation of skeletal muscle EMP into plasmablastic plasmacytoma. In this article, we report it in order to improve clinicians' awareness of the diagnosis, treatment and prognosis of this pathologic transformation case, and to avoid misdiagnosis and underdiagnosis.

## Case presentation

**Fig. 1:**
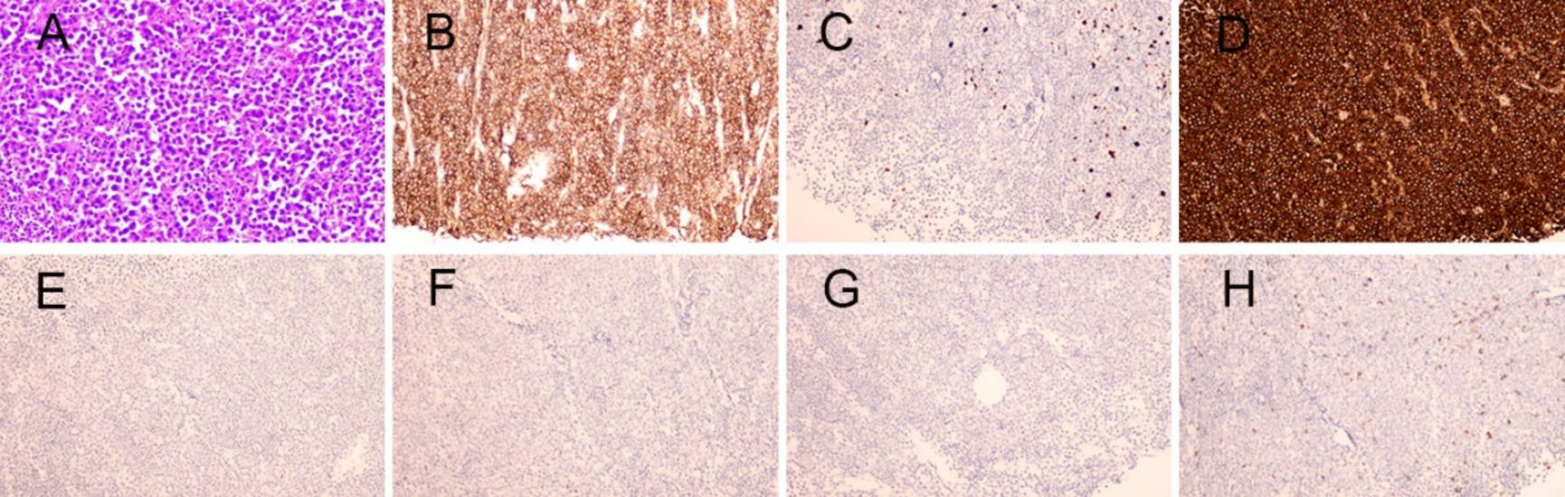
**A** The tumor cells morphologically resemble mature plasma cells, with rounded nuclei and abundant cytoplasm. (hematoxylin and eosin × 200); Immunohistochemical staining showed **B** expression of CD138, **C** Ki-67 index is ~ 10%, **D** kappa light chain positivity, **E** absence of lambda light chain expression, and **F** absence of CK, **G** CD20, **H** CD3

A 70-year-old man was hospitalized on January 16, 2020, with a mass in his right calf that had been swollen and painless for 6 months. On physical examination, the mass was approximately 4 cm × 14 cm in size. X-rays showed a soft tissue mass on the lateral side of the right tibia and fibula. A whole-body bone scan showed no significant concentration of abnormal bone nuclides. To clarify the nature of the mass, a biopsy of the right gastrocnemius mass was performed, and pathology showed non-Hodgkin's lymphoma (Fig. 1A). Further Immunohistochemical staining showed that CD138 ( +),CK ( – ),CD3 ( – ), CD20 ( – ),κ( +)/λ( – ), and Ki-67 were about 10%, which was consistent with the EMP (Fig. 1B–H). In addition, blood analysis, biochemical tests, and blood immunoglobulin tests were normal, and the urinary Bence-Jones protein was negative, and bone marrow examination showed no abnormality. No distant metastasis on whole-body CT scan. The whole-body bone scan was normal. The patient’s EpsteinBarr Virus (EBV) and Human Immunodeficiency Virus (HIV) test results were negative. Serum protein electrophoresis showed no significant abnormalities and Immunoelectrophoresis negative. κ-FLC: λ-FLC (rFLC) is normal in serum and urinary (Table 1). Clinical diagnosis: gastrocnemius EMP. The patient received sequential chemoradiotherapy. On January 30, 2020, due to the large size of the mass, the patient received one cycle of PAD (bortezomib, 1.3 mg/m^2^ on days 1, 4, 8, and 11; doxorubicin, 10 mg/m^2^ on days 1–4; dexamethasone, 20 mg on days 1, 2, 4, 5, 8, 9, 11, and 12, every 21 days) chemotherapy, and the mass subsided with efficacy evaluation as a partial response. The patient underwent local radiotherapy from February 2020 to March 2020. The total radiation dose was 56 Gy, given in 28 fractions. Subsequently, 5 cycles of PAD chemotherapy were given, the tumor disappeared, and an efficacy evaluation of complete remission.

**Table 1:** All electrophoresis and free light chain results for the patient

	First diagnosis	Relapse	Second relapse	Reference interval
Serum electrophoresis				
ALBUMIN	57.3	62. 1	62. 6	59. 8–72.4 (%)
ALPHA 1	4. 1	3.6	4.5	1.0–3.2 (%)
ALPHA 2	12. 2	10. 9	14. 1	7. 4–12.6 (%)
BETA 1	5. 3	4. 7	5. 6	4. 7–7. 2 (%)
BETA 2	5. 6	4.6	4. 0	3.2–6.5 (%)
GAMMA	15.5	14. 1	9. 2	8. 0–15. 8 (%)
A/G	1. 34	1.64	1. 67	
Serum free light chain				
κ-FLC	9.32	24.31	5.47	3.30–19.40 (mg/L)
λ-FLC	10.11	22.48	6.21	5.71–26.30 (mg/L)
rFLC	0.9219	1.0814	0.8808	0.26–1.65
dFLC	0.79	1.83	0.74	(mg/L)
Urinary free light chain				
κ-FLC	4.88	11.34	3.77	0.00–25.80 (mg/L)
λ-FLC	2.10	2.82	0.72	0.00–11.30 (mg/L)
rFLC	2.3238	4.0213	5.2361	1.40–6.20
dFLC	2.78	8.52	3.05	(mg/L)

rFLC, κ-FLC: λ-FLC; dFLC, | κ-FLC-λ-FLC |

**Fig. 2:**
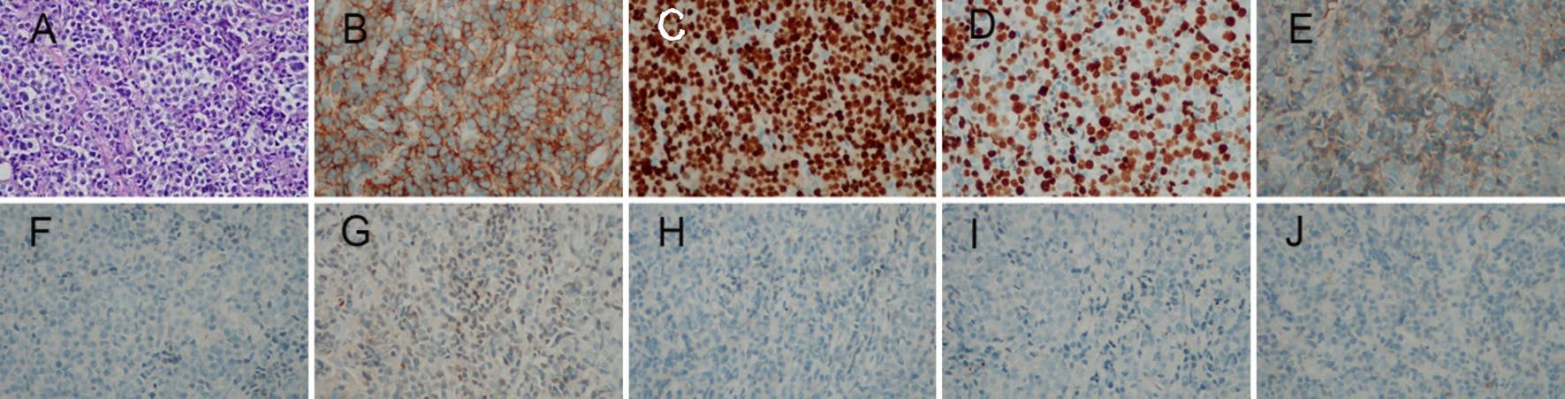
**A** The tumor cells are round or oval, with large nuclei, pronounced nucleoli, and immunoblast-like morphology (hematoxylin and eosin × 200); Immunohistochemical staining showed **B** expression of CD138, **C** MUMI, **D** Ki-67 index is ~ 60%, **E** kappa light chain positivity, **F** absence of lambda light chain expression, and absence of **G** Bcl-6, **H** CK, (**I**) CD20, **(J**) CD3

**Fig. 3:**
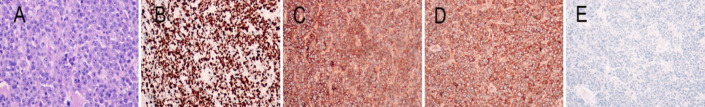
**A** The tumor cells are large, with eccentric nuclei and basophilic cytoplasm. (hematoxylin and eosin × 200); Immunohistochemical staining showed **B** Ki-67 index is ~ 60%, **C** expression of CD138, **D** kappa light chain positivity, and **E** absence of lambda light chain expression

Six months later, Patient returned for progressive enlargement of a mass on the right inner thigh. On physical examination, a mass measuring approximately 4 cm × 5 cm was touched, with hard, clear margins and no tenderness on the right inner thigh. In addition, a mass measuring approximately 2.5 cm × 3.5 cm was touched on the right lower abdominal wall, which was hard, with indistinct margins and no tenderness. CT scan showed multiple soft tissue density mass shadows within the right lower abdominal wall adipose tissue and right posterior medial femoral muscle group. Histological examination of the subcutaneous mass was performed: the tumor cells were round or oval, with large nuclei, pronounced nucleoli, and immunoblast-like morphology (Fig. 2A). Immunohistochemistry showed CD138 ( +), Bcl-6 ( – ), CD20 ( – ), CD3 ( – ), κ ( +)/λ ( – ), MUM1 ( +), CK ( – ), and a high Ki-67 labeling index of about 60% (Fig. 2B–J). in situ hybridization of small mRNA encoded by EBV (EBER) was negative. bone marrow biopsy showed active bone marrow proliferation without abnormal plasma cells. EBV and HIV test results were negative. Serum protein electrophoresis showed no significant abnormalities and Immunoelectrophoresis negative. κ-FLC: λ-FLC (rFLC) is normal in serum and urinary (Table 1). Repeat whole-body bone imaging showed no abnormality. This indicated that the EMP was transformed into plasmablastic plasmacytoma. From February 2021 to April 2021 received radiotherapy. Concurrently, the patient received 2 cycles of VRD (bortezomib, 1.3 mg/m^2^ on days 1, 4, 8, and 11; lenalidomide, 25 mg on days 1–14; and dexamethasone, 20 mg on days 1, 2, 4, 5, 8, 9, 11, and 12, every 21 days) chemotherapy. For the right inner thigh tumor, 6 MV X-rays were given at a total dose of 54 Gy in 27 fractions; the right abdominal wall tumor was given 6 MV X-rays, 30 Gy in 15 fractions followed by 8 MeV electron beam irradiation, 24 Gy in 12 fractions for a total dose of 54 Gy. The subcutaneous tumor disappeared after treatment. However, 2 months later, Patient presented with a painless mass found in the left lower extremity and cough and sputum. On physical examination, a mass measuring about 7 cm × 8 cm was touched on the lateral side of the left lower extremity, with medium texture, poor mobility, no pressure pain, and no redness or swelling on the skin surface. Combined chest, abdomen, and pelvis CT showed multiple metastases in the lungs, right axillary lymph nodes, and left lower extremity. A third biopsy of the subcutaneous mass was performed, and pathologic examination showed PBM (Fig. 3A). Immunohistochemistry results: CD138 ( +), κ ( +)/λ ( – ), and a high Ki-67 labeling index of about 60% (Fig. 3B–E). bone marrow biopsy showed active bone marrow proliferation without abnormal plasma cells. Repeat whole-body bone imaging showed no abnormality. EBV and HIV test results were negative. Serum protein electrophoresis showed no significant abnormalities and Immunoelectrophoresis negative. κ-FLC: λ-FLC (rFLC) is normal in serum and urinary (Table 1). From July 2021 to October 2021, chemotherapy was changed to the DPD (daratumumab, 16 mg/kg weekly; pomalidomide, 4 mg on days 1–21; and dexamethasone, 20 mg on days 1,2,8,9,15,16, 22and 23, every 28 days) greimen for 3 cycles. Subcutaneous metastases were smaller than before, but new metastases were found in the pelvic cavity, bilateral inguinal areas, and adrenal glands. In November 2021, the chemotherapy regimen was changed to DECP (dexamethasone 20 mg on days 1–4; etoposide, 100 mg/d on days 1–3; cyclophosphamide, 500 mg/m^2^ on days 1–4; and cisplatin, 20 mg/m^2^ on days 1–3, every 28 days), but there was no significant effect and the disease continued to progress. Eventually, the patient developed pneumonia and died of respiratory failure. Overall survival was 22 months, only 8 months after the pathologic type was transformed to plasmablastic plasmacytoma.

## Discussion and conclusion

EMP is a plasma cell tumor that occurs outside the bone marrow hematopoietic tissue. Most of the affected individuals are men, the ratio of male to female is about 2∶1, and the median age of onset is 55–60 years (Zhu et al. 2020). The clinical symptoms of EMP are directly related to the site of tumor infiltration, with 80–90% of EMPs occurring in the head and neck region, and most commonly in the nasal cavity, sinuses, and nasopharynx. Regardless of where it occurs, EMP is a space-occupying lesion that presents with nonspecific symptoms in localized organs. In the nasal cavity, the mass mainly presents as polypoid hyperplasia or diffuse submucosal thickening, dark red or grayish-yellow in color, with a smooth surface and medium-hard texture (Hu et al. 2020). When the lesion is located in the pharynx, patients present with throat discomfort and hoarseness. Tumors often appear as polyps or nodules, some with ulcers and surface bleeds easily (Tang et al. 2019). Other sites include the gastrointestinal tract, lungs, lymph nodes, spleen, skin, subcutaneous tissue, thyroid gland and ureter, but they are rare (Gupta et al. 2022; Okada et al. 2021; Wang et al. 2009; Wang and Xiao 2022). Our patient, an elderly male, had a primary site in the gastrocnemius with no obvious clinical symptoms, it presented only as a swelling of the right calf without pain. Before the diagnosis was confirmed by pathologic examination, it was easy to be misdiagnosed as muscle soft tissue inflammation or lymphoma.

Currently, the following criteria are commonly used for the diagnosis of EMP internationally: (1) biopsy-proven extramedullary monoclonal plasma cell tumor; (2) normal bone marrow with no evidence of clonal plasma cells or clonal bone marrow plasma cells < 10%; (3) normal skeletal examination and magnetic resonance imaging (or computed tomography) of the spine and pelvis (except for primary solitary lesions); (4) no damage to internal organs, such as hypercalcemia, renal damage, anemia, and bone lesions (CRAB) attributable to lymphoplasmacytic proliferation; (5) no or low serum M protein concentrations (Rajkumar et al. 2014). Microscopically, the EMP consists of sheets of plasma cells of varying degrees of differentiation, and the grading of the tumor can be highly variable. In poorly differentiated tumors, the cellular heterogeneity is obvious, the nucleus is enlarged, the prominent nucleoli, the nuclear schizophrenia is common, and it seems to be plasma mother or immunoblast-like morphology (Meyer et al. 2018). Immunohistochemistry of the EMP shows light chain expression, that is κ( +), λ( – ), or κ( – ), λ( +), and expression of CD138 and or CD38 (Firsova et al. 2020). The clinical laboratory tests and pathologic biopsy results of our patient met the diagnostic criteria for EMP. Due to the low incidence of EMP and the lack of specific clinical symptoms and imaging features, it needs to be differentiated from lymphoplasmacytic lymphoma (LPL) and MM in diagnosis. LPL is a mixture of small B-lymphocytes, lymphoplasmacytic cells and monoclonal plasma cells with an immunohistochemical lymphoid component that expresses CD20, but not CD138, and is usually associated with EBV infection (Fend et al. 2023). This patient’s microscopic presentation and immunohistochemical findings were inconsistent with those of LPL. Since EMP has the potential to progress to MM, MM must be excluded in patients with a proposed diagnosis of EMP. It can be differentiated from EMP by CRAB characteristics (hypercalcemia, renal damage, anemia, and bone lesions), bone marrow biopsy, and M protein levels in blood or urine (Tyczyńska et al. 2023). In this paper we report a case of EMP originates in the gastrocnemius and transformed into plasmablastic plasmacytoma. Plasmablastic plasmacytoma is a highly malignant tumor with a poor prognosis. The morphologic features of PBM are the presence of large plasma cells with large, darkly stained nuclei; one or more distinct, centrally located nucleoli; cytoplasmic basophilic or bichromophilic; a high nucleoplasmic ratio; an increase in mitotic counts; and Immunohistochemistry is characterized by the expression of plasma cell-associated antigens, including MUM1/IRF4, CD138 and CD38, light chain restricted expression and high Ki-67 expression, but not CD20, Bcl-6, PAX5 (Mori et al. 2021). Plasmablastic plasmacytoma needs to be differentiated from plasmablastic lymphoma (PBL). Morphologically, tumor cells of PBL resemble immunoblasts, plasmoblasts or with plasma-like differentiation, but nuclear schizophrenia is easily seen and apoptosis and necrosis are often present. Immunohistochemically, PBL also expresses plasma cell-associated antigens and usually not CD45 and B-cell-associated antigens; most have Ki-67 > 90% (Bailly et al. 2022). Vega’s (Vega et al. 2005) study showed that EBER is positive in all PBL but negative in plasmablastic plasmacytoma. Currently, EBER is the most useful marker to differentiate between PBL and PBM (Ahn et al. 2017). PBL is a less common type of large B-cell lymphoma that occurs in patients with immunodeficiencies, such as acquired immunodeficiency syndromes, the first site is often the oral cavity or gastrointestinal tract, and 76% of them are infected with EBV (Bailly et al. 2022). Our patient, who had no history of immunodeficiency and was negative for HIV, EBV and EBER, was diagnosed with EMP. After metastasis occurred, a combination of two pathologic and immunohistochemical findings led to the diagnosis of EMP transformed into plasmablastic plasmacytoma. The diagnosis of this disease is clinically instructive because it has never been reported before.

At present, the main treatment modalities for EMP include surgery, radiotherapy, chemotherapy and combination therapy. Scholars at home and abroad believe that due to the high radiosensitivity of EMP, radiotherapy alone can achieve satisfactory efficacy, and radiotherapy should be preferred for its treatment (Tsang et al. 2018). Radiotherapy irradiation should cover the full extent of the lesion, as shown on magnetic resonance imaging and computed tomography, and include a certain area of surrounding healthy tissue. The dose of radiotherapy is usually 40 Gy-60 Gy, and the course of treatment is 4–6 weeks (Sanchez et al. 2023). However, the choice of treatment should be based on the location and extent of the lesion. When the lesion is relatively limited and has enough resection area, it can be treated by surgery alone, such as when the tumor is in the thyroid gland, cervical lymph nodes, gastrointestinal tract (Gilder et al. 2018), Adjuvant chemotherapy is considered for patients with high-risk factors, such as tumor diameter > 5 cm, poor differentiation, or the disease is difficult to control (Holler et al. 2022). Commonly used chemotherapeutic agents include melphalan, dexamethasone, adriamycin and cyclophosphamide. With the advent of new systemic chemotherapeutic agents, including proteasome inhibitors (bortezomib, ixazomib, and carfilzomib), immunomodulators (thalidomide, lenalidomide, and pomalidomide), and monoclonal antibodies (daratumumab, Isatuximab, and elotuzumab) (Oka et al. 2020). In this case, the tumor was located in the gastrocnemius and had a large diameter, we gave sequential radiotherapy and chemotherapy to the lesion. Radiotherapy dose of 56 Gy, combined with the PAD regimen, resulted in significant regression of the mass, with a duration of response of approximately 6 months. Subsequently, the EMP progressed and transformed into plasmablastic plasmacytoma. Due to the rarity of plasmablastic plasmacytoma, there is currently no standard treatment regimen. We attempted to give the patient a radiotherapy dose of 54 Gy to the lesions on the right medial thigh and right lower abdominal wall in combination with chemotherapy in VRD regimen, and the lesions disappeared, obtaining a complete remission without any significant radiological damage. It has been reported that plasmablastic plasmacytoma may be associated with increased levels of vascular endothelial growth factor expression (Chung and Liedtke 2019), so when the disease progressed again, we tried the DPD and DECP regimens, but none of them showed significant efficacy, and the patient eventually died of respiratory failure. This confirms the high sensitivity of plasmablastic plasmacytoma to radiotherapy and may provide a new way of thinking about the treatment of PBM.

EMP has a relatively good prognosis, with a 10-year survival rate of more than 70% (Janjetovic et al. 2021), but some patients have localized recurrence or progression to MM with a relatively poor prognosis. In this case, after pathologic to plasmablastic plasmacytoma, the disease progressed rapidly and the overall survival was only 22 months, which was much lower than that of most EMPs. This also confirms that plasmablastic plasmacytoma is a highly malignant tumor with poor prognosis and short survival.

## Data Availability

All data generated or analyzed during this study are included in this published article.

## Abbreviations

EMP: Extramedullary plasmacytoma
SBP: Solitary bone plasmacytoma
MM: Multiple myeloma
EBV: Epstein-Barr Virus
HIV: Human Immunodeficiency Virus
PAD: Bortezomib doxorubicin dexamethasone
EBER: Small mRNA encoded by EBV
VRD: Bortezomib lenalidomide dexamethasone
DPD: Daratumumab pomalidomide dexamethasone
DECP: Dexamethasone etoposide cyclophosphamide cisplatin
CRAB: Hypercalcemia, renal damage, anemia, and bone lesions
LPL: Lymphoplasmacytic lymphoma
PBL: Plasmablastic lymphoma
rFLC κ-FLC: λ-FLC
dFLC: | κ-FLC-λ-FLC |
